# Factors Influencing Burnout Syndrome in Obstetrics and Gynecology Physicians

**DOI:** 10.1155/2017/9318534

**Published:** 2017-12-05

**Authors:** Magdalena Iorga, Vladimir Socolov, Diana Muraru, Catalin Dirtu, Camelia Soponaru, Ciprian Ilea, Demetra-Gabriela Socolov

**Affiliations:** ^1^Behavioral Sciences Department, University of Medicine and Pharmacy “Gr. T. Popa”, Iași, Romania; ^2^Department of Obstetrics and Gynecology, University of Medicine and Pharmacy “Gr. T. Popa”, University Maternity Hospital “Cuza Voda”, Iași, Romania; ^3^Department of Preventive Medicine, University of Medicine and Pharmacy “Gr. T. Popa”, Iași, Romania; ^4^Faculty of Psychology and Educational Sciences, University “Alexandru Ioan Cuza”, Iași, Romania; ^5^Department of Obstetrics and Gynecology, University of Medicine and Pharmacy “Gr. T. Popa”, Iași, Romania

## Abstract

**Aim:**

To identify the influence of environmental, personality, and alexithymia factors on burnout syndrome among obstetrics and gynecology physicians.

**Material and Methods:**

A total of 116 physicians (35 men and 81 women) completed questionnaires and sociodemographic data sheets. The* Maslach Burnout Inventory,* the* Big Five Inventory*, and* the Toronto Alexithymia Scale* were used to measure burnout, innate personality traits, and alexithymia, respectively. The *t*-test and Pearson correlations were used for other measurements.

**Results:**

Of the 116 study physicians, 12.9% have suffered or still suffer from depression and 35.3% have had or still have problems related to insomnia. Regarding emotional exhaustion and depersonalization factors, men obtained lower scores than women (18.73 ± 13.48 versus 24.14 ± 11.71 for emotional exhaustion; 5.97 ± 5.45 versus 7.70 ± 5.29 for depersonalization). Self-reported depression was related to higher scores for all 3 domains, to higher total scores for alexithymia and neuroticism, and to lower scores for extraversion, conscientiousness, and openness. Residents and consultants had markedly different scores.

**Conclusion:**

The results of this study will help obstetrics and gynecology physicians cope with professional burnout and to consider personality traits, alexithymia domains, and environmental factors when finding strategies to cope with their professional stress.

## 1. Introduction

The practice of medicine is characterized by a great deal of humanism. The care of patients demands a lot of hard work, patience, empathy, physical and psychological resistance against stressful factors, and working under a lot of pressure. These aspects have an impact on physicians' personal and professional lives.

Burnout has been defined by Maslach et al. [1] as a syndrome consisting of emotional exhaustion, depersonalization (i.e., a tendency to have negative and cynical thoughts towards other people, patients included), and a reduced sense of personal achievement. Burnout differs from the global sense of depression, because it refers to work-related exhaustion. High levels of burnout and depersonalization, in addition to a low level of personal achievement, affect the quality of medical performance, interaction with the patient, and physicians' quality of life.

Much research has focused on burnout syndrome among healthcare professionals to identify predictors, such as personality traits, extraversion, neuroticism, and work-related environmental stressors, such as the type of medical unit and the relationship with colleagues or superiors, and also personal issues related to the relationship with a partner, as well as other personal issues [2–7].

Burnout syndrome has been seen as an important concern among medical professionals, because it is related to low quality of care for patients. Studies show that, compared with the general population, physicians experience a high level of burnout, dissimilar to other professions [8]. Burnout is associated with an increased error rate, suicidal thoughts and suicide, a difficult relationship with a life partner, and substance abuse, including alcohol consumption [9]. Surgeons, intensive care physicians, and anesthesiologists have high burnout scores. Men, residents, and surgeons are more prone to burnout [10–13].

Apart from environmental factors (occupation, workplace, type of medical interventions, hours worked per week, and others), personality factors are closely connected with a predisposition towards burnout (neuroticism and extraversion).

Few studies have focused on identifying the level of burnout and its relation to personality factors in obstetrics and gynecology physicians. The research objectives are identifying (a) the influence of environmental factors (sex, age, marital status, number of children, years of professional experience, hours of work per week, teaching level, workplace, number of shifts per week, number of positions, the presence or absence of insomnia, the presence or absence of depression, the presence of chronic diseases, and the use of antistress pills) on burnout (emotional exhaustion, depersonalization, and personal achievement) and (b) the influence of Big Five personality factors (extraversion, neuroticism, openness, consciousness, and agreeableness) on the level of burnout.

## 2. Material and Methods

The study sample consisted of 220 obstetrics and gynecology physicians who work in hospitals, clinics, or private medical practices and who were asked to participate in research to identify the presence of burnout syndrome and job satisfaction in this medical specialty. A total of 116 physicians (35 men and 81 women) completed the study questionnaires and sociodemographic data sheet. The questionnaires were self-administered by the subjects. For physicians who live in other cities in the country, the questionnaires were sent by mail, and for physicians who live at the university center where the research was carried out, the questionnaires were distributed by the investigators and returned to them, after being filled in. Informed consent was obtained from participants. Each questionnaire had a separate informed consent sheet, detailing information about the research objective, data privacy, and the fact that the results were only going to be used for this study.

Burnout among physicians was measured using the* Maslach Burnout Inventory* (MBI), a validated 22-item questionnaire that is the most frequently used tool for measuring burnout [1]. The MBI has 3 subscales to evaluate each domain of burnout, which include (1) emotional exhaustion (describing the feeling of being exhausted and drained by one's work, fatigued at the very idea of work, chronic fatigue, trouble sleeping, and physical problems), (2) depersonalization (describing emotional coldness and impersonal reactions to the beneficiaries of one's work, leading to cynicism and negative attitudes with regard to patients or colleagues, feelings of guilt, avoidance of social contacts, and withdrawal into oneself), and (3) low personal achievement (describing feelings of competence and accomplishment in one's work with other people; the demotivating effects of a difficult, repetitive situation leading to failure despite one's effort. The person begins to doubt his genuine abilities to accomplish things. This aspect is a consequence of the first two. The items of this scale are written as assertions about personal feelings and attitudes). A high degree of burnout is reflected in high scores on the emotional exhaustion and depersonalization subscales and low scores on the personal accomplishment subscale. An average degree of burnout is reflected in average scores on the 3 subscales. A low degree of burnout is reflected in low scores on emotional exhaustion and depersonalization subscales and in high scores on the personal accomplishment subscale [14].

Burnout scores are as follows: emotional exhaustion: ≤16: low-level burnout; 17 to 26: moderate burnout; ≥27: high-level burnout. Depersonalization scores are as follows: ≤6: low-level burnout; 7 to 12: moderate burnout; ≥13: high-level burnout. Personal achievement scores are as follows: ≤31: high-level burnout; 32 to 38: moderate burnout; ≥39: low-level burnout.

The* Big Five Inventory* (BFI) [15, 16] measures innate personality traits. The questionnaire consists of 44 items corresponding to the 5 personality factors of the Big Five model: extraversion, neuroticism, openness, consciousness, and agreeableness. The answers were offered by subjects on a Likert-like scale with 5 steps, where 1 means strong disagreement and 5 means strong agreement.

*The Toronto Alexithymia Scale (TAS-20)* is a 20-item self-administered scale used to measure alexithymia, a psychological construct that refers to people who have trouble identifying and describing emotions and who tend to minimize emotional experience, because they focus their attention externally [17].

Other variables were considered and registered, including age, level of specialization (resident, specialist, or primary doctor), department, environment (urban/rural), marital status, data about partnership (the life partner has/does not have the same occupation or specialty, if the partner is also a physician), information about the family of origin (number of siblings), number of children, number of years of experience in obstetrics and gynecology, involvement in academic activities, the type of institution (hospital, private clinic, and private medical practice), the number of current jobs, the number of hours worked per week, and the number of shifts per month. Some questionnaire items requested information about insomnia, the use of drugs to cope with stress, depressive periods in the past or present, and known chronic diseases.

Data analysis was performed using IBM SPSS Statistics version 23. For the comparative analysis, the *t*-test for independent samples and one-way ANOVA were used. Statistical significance was defined as *p* < .05. For the correlational study, we used Pearson and Spearman correlations, and to identify the influence of personality and alexithymia factors on burnout factors, we used multiple linear regressions.

## 3. Results

### 3.1. Descriptive Analysis

#### 3.1.1. Instruments

For MBI, Cronbach's alpha coefficients obtained in the 3 scales were 0.911, 0.630, and 0.816, respectively. For the BFI, we obtained the following results: extraversion (8 items, Cronbach's alpha coefficient 0.802), agreeableness (9 items, Cronbach's alpha coefficient 0.467), conscientiousness (9 items, Cronbach's alpha coefficient 0.772), neuroticism (8 items, Cronbach's alpha coefficient 0.769), and openness to experience (10 items, Cronbach's alpha coefficient 0.743). Despite the poor reliability for the agreeableness dimension (a Cronbach's alpha score of 0.467), we included this score in our study. One of the causes for this low score is the fact that the majority of variables are not normally distributed. To remedy this low reliability, we first inspected the Interitem Correlation Matrix (higher scores were obtained for some items and lower scores for others, explaining the medium score for Cronbach's alpha total score). The second solution proposed is to inspect the Intertotal Statistics Table, the column of Cronbach's alpha if items were deleted. This is the second statistical measure that we considered (if we eliminated one item, the score for Alpha Cronbach would be 0.650 [acceptable]). But some authors considered it to be a positive solution to keep the item even if the value was low in case of an item with an important role, which is the case for our instrument.

For measuring alexithymia, we used the third instrument, the Toronto Alexithymia Scale (TAS-20), with 3 subscales: difficulty identifying feelings or emotions (Cronbach's alpha coefficient 0.749), difficulty describing feelings or emotions (Cronbach's alpha coefficient 0.659), and externally oriented thinking, which refers to people's tendency to focus their attention externally (Cronbach's alpha coefficient 0.597), and the total coefficient for alexithymia (Cronbach's alpha is 0.871).

#### 3.1.2. Sociodemographic and Psychological Data

The subjects were from 10 counties, 5 of which have university centers with faculties of medicine in Romania. The number of registered physicians in 2016 was 1724 (working for public and private institutions) and 693 physicians working exclusively in private clinics. Therefore, the physicians included in the study represent 6.72% of all registered obstetrics and gynecology physicians in Romania. The higher percentage of female physicians is specific to the general population in this specialty.

The following variables were considered: age, sex, level of specialization (resident, specialist, or primary doctor), work environment (urban, rural), number of siblings, and number of children. The physicians were also surveyed regarding their marital status and their partner's occupation to find out if these variables influenced burnout or professional satisfaction, taking into account the fact that understanding the difficulties related to this profession could help the subject adequately adapt to stressors.

A total of 42 residents, 11 specialists, and 63 consultant physicians were surveyed. Their ages ranged between 25 and 68 years, and they came from 10 departments (covering approximately one-fourth of the national territory).

A total of 91 subjects declared they are or have been in a relationship (they are married, in a partnership, or divorced). Of these, 45 (almost half) have or have had a physician as a partner, thus, a partner with the same occupation. Of these 45, a total of 15 declared that the partner was in the same medical specialty. We have also taken into account variables related to professional activity: years of professional experience, hours of work per week, number of shifts per month, teaching activity (if the subjects are academics), and the number of current jobs. In addition, certain items have questioned the physicians on whether they use pills to cope with stress, whether they have suffered or suffer from insomnia or depression, and whether they suffer from chronic diseases. The data obtained are presented in Table 1. The above-mentioned variables were considered in order to identify further whether there is a significant connection between them and the levels of burnout, depersonalization, and personal achievement.

The physicians were questioned regarding whether they used pills to cope with stress at work; 12 (10.3%) claimed that they did. Moreover, 2 items addressed the presence of depression and insomnia. A total of 15 (12.9%) physicians claimed that they had suffered or still suffered from depression, and 41 (35.3%) declared that they had or still had problems related to insomnia. So, over one-third of doctors reported having sleep disorders.

Almost 40% of physicians declared that they suffer from chronic diseases. The most frequently mentioned health problems were chronic hypertension, migraines, herniated disc, and lumbosciatica. These chronic diseases are related to the postural position during investigation, treatment, and surgical interventions and also are determined by the high number of hours of sleep deprivation.

General scores for MBI, BFI, and TAS-20 for male and female subjects are presented in Table 2.

Results for general scores for MBI for all 3 domains reflect an average level of burnout among obstetrics and gynecology physicians. Regarding the emotional exhaustion and depersonalization factors, men had lower scores compared with women (emotional exhaustion, 18.73 ± 13.48 versus 24.14 ± 11.71; depersonalization, 5.97 ± 5.45 versus 7.70 ± 5.29).

The total alexithymia score reflects a low level of alexithymia in both male and female participants. TAS-20 used cutoff scoring as follows: ≤51 indicates nonalexithymia; ≥61 indicates alexithymia. Scores of 52 to 60 indicate possible alexithymia.

### 3.2. Comparative Analysis

#### 3.2.1. Sociodemographic and Psychological Data

To identify whether there are significant differences in values for burnout, in BFI and TAS-20 domains, depending on subjects' sex, we used the *t*-test for independent samples. There were significant differences between subjects' sex and emotional exhaustion (*t*(113) = −2.149, *p* = .034, Mfemale = 24.14 and Mmale = 18.76). No other statistically significant differences were observed regarding other variables.

We used one-way ANOVA. According to the level of specialization, significant differences were identified regarding depersonalization between residents and consultants (*F*(2.113) = 3.512, *p* = .033 with Md = 2.730, *p* = .030), meaning that residents obtained significantly higher scores in depersonalization compared with consultants. A significant difference was also registered between these two categories for personal achievement (*F*(2.113) = 7.879, *p* = .001 with Md = −5.72, *p* = .001), meaning that the level for personal achievement is higher in primary doctors. Results for the 3 domains of MBI by level of specialization are presented in Table 3.

Regarding alexithymia scores, considering the level of specialization, no differences were found regarding difficulty describing feelings and externally oriented thinking, or in total score. In difficulty identifying feelings, residents have obtained statistically significantly higher scores than consultants (*F*(2.112) = 3.310, *p* = .040 with Md = 3.10, *p* = .037).

For BFI domains, only agreeableness was identified as significantly different between specialists and consultants, meaning that the first category of doctors has higher scores compared with consultants (*F*(2.113) = 3.195, *p* = .045 with Md = .669, *p* = .041).

Our research also seeks to identify whether having a physician as life partner had an effect on the level of burnout and professional achievement, because almost 40% of the questioned subjects have a partner in the same profession. No significant differences were identified between physicians who had physician life partners, compared with those whose partners had a different profession regarding the considered variables.

According to marital status and teaching experience, no significant influences impacted the level of burnout or alexithymia.

#### 3.2.2. Insomnia, Depression, Pills, and Chronic Disease

Statistical processing (independent-samples *t*-test) emphasized the effects of insomnia on emotional exhaustion (*t*(114) = 4.265, *p* = .000, Mwith = 28.70 and Mwithout = 19.12) and depersonalization (*t*(114) = 3.176, *p* = .002, Mwith = 9.24 and Mwithout = 6.06). The people who suffered from insomnia obtained higher scores in emotional exhaustion and depersonalization compared with subjects without insomnia. Those claiming that they do not suffer from insomnia had higher levels for personal achievement (*t*(114) = −2.813, *p* = .006, Mwith = 34.04 and Mwithout = 38.20).

Insomnia also had a significant influence on two of the alexithymia factors (difficulty describing feelings (*t*(113) = 3.545, *p* = .001, Mwith = 13.82 and Mwithout = 11.16) and difficulty identifying feelings (*t*(113) = 5.428, *p* = .000, Mwith = 19.63 and Mwithout = 13.74)) and on total score (*t*(113) = 4.222, *p* = .000, Mwith = 51.68 and Mwithout = 42.54).

We also wanted to see if there are any differences considering the insomnia variable and personality traits measured by BFI. Significant differences between subjects with and without self-declared insomnia were identified, related to extraversion (*t*(114) = −3.806, *p* = .000, Mwith = 3.39 and Mwithout = 3.89), neuroticism (*t*(114) = 5.202, *p* = .000, Mwith = 3.22 and Mwithout = 2.56), agreeableness (*t*(114) = −1.944, *p* = .054, Mwith = 3.64 and Mwithout = 3.96), and conscientiousness (*t*(114) = −2.193, *p* = .030, Mwith = 3.83 and Mwithout = 4.08). The results prove that people with insomnia have higher scores for neuroticism and lower scores for extraversion, agreeableness, and conscientiousness.

There are statistically significant differences between the mean values of subjects with and without depression regarding emotional exhaustion (*t*(113) = 3.180, *p* = .002, Mwith = 31.60 and Mwithout = 21.06). For depersonalization, significant differences were also obtained (*t*(113) = 2.160, *p* = .033, Mwith = 9.93 and Mwithout = 6.76). For the personal achievement domain, results are significant considering this variable (*t*(113) = −2.681, *p* = .015, Mwith = 31.86 and Mwithout = 37.47). The results prove that physicians with self-declared presence of depression obtained higher scores for emotional exhaustion and depersonalization and lower scores for personal achievement.

Regarding alexithymia, the presence of self-reported depression is related to higher scores for all 3 domains and total score of alexithymia: *t*(112) = 2.949, *p* = .004, Mwith = 14.93 and Mwithout = 11.72 for difficulty describing feelings; *t*(112) = 3.524, *p* = .001, Mwith = 20.93 and Mwithout = 15.13 for difficulty identifying feelings; *t*(112) = 2.944, *p* = .004, Mwith = 21.06 and Mwithout = 17.39 for externally oriented thinking. For total score of alexithymia, people who declare being depressed had higher total scores (*t*(112) = 4.109, *p* = .000, Mwith = 56.93 and Mwithout = 44.25).

We compared subjects with and without depression in relation to BFI domains. We found that depression determines statistically significant differences for the following factors of BFI: extraversion (*t*(113) = −4.190, *p* = .000, Mwith = 3.03 and Mwithout = 3.81), conscientiousness (*t*(113) = −3.055, *p* = .005, Mwith = 3.69 and Mwithout = 4.03), neuroticism (*t*(113) = 2.615, *p* = .010, Mwith = 3.23 and Mwithout = 2.72), and openness (*t*(113) = −1.982, *p* = .050, Mwith = 3.32 and Mwithout = 3.64). The results show that people with self-declared depression have higher scores for neuroticism and lower scores for extraversion, conscientiousness, and openness.

The statistical data analysis identified that the levels for burnout domains, alexithymia factors, or total score were not influenced by the presence of drug consumption to face daily stress or by the presence of a chronic disease. Subjects who took pills to cope with stress had lower scores for extraversion (*t*(113) = −3.362, *p* = .001, Mwith = 3.09 and Mwithout = 3.79). That means that introverts are more likely to use pills for coping with daily distress.

### 3.3. Correlational Analysis

To perform the correlational analysis and to know which tests should be used for statistical analysis, we first tested the normality of our data distribution. For this, the Kolmogorov-Smirnov test was used for all the variables investigated. The total score of the Toronto Alexithymia Scale was K-S *z* = 0.076, *p* = .100, and because *p* > 0, the scores are considered normally distributed. TAS-20 scores were for difficulty describing feelings K-S *z* = 0.100, *p* = .006, difficulty identifying feelings K-S *z* = 0.079, *p* = .074, and externally oriented thinking K-S *z* = 0.069, *p* = .200. For almost all the factors of the TAS-20, the score distributions were normal, except for the difficulty identifying feelings, because *p* < .05.

MBI scores were as follows: emotional exhaustion K-S *z* = 0.077, *p* = .091, depersonalization K-S *z* = 0.114, *p* = .001, and personal accomplishment K-S *z* = 0.102, *p* = .005. The scores are normally distributed only for emotional exhaustion, for a *p* > .05. For depersonalization and personal achievement, scores are not normally distributed, with a *p* < .005.

For BFI factors, the results were extraversion K-S *z* = 0.068, *p* = .200, agreeableness K-S *z* = 0.140, *p* = .000, conscientiousness K-S *z* = 0.101, *p* = .005, neuroticism K-S *z* = 0.078, *p* = .081, and openness K-S *z* = 0.090, *p* = .022. The results prove that extraversion and neuroticism are normally distributed, but agreeableness, openness, and consciousness are not.

For the other sociodemographic variables in this correlational analysis, the following results were found: age: K-S *z* = .145, *p* = .000; the number of children: K-S *z* = .237, *p* = .000; years of experience: K-S *z* = .159, *p* = .000; working hours per week: K-S *z* = .100, *p* = .009; and shifts per month: K-S *z* = .146, *p* = .000. These variables were not distributed normally; therefore, Spearman correlation, a nonparametric test, was used for correlational analysis.

Pearson correlation coefficient was used for normally distributed variables, and Spearman rank correlation was used for the scores that are not distributed normally. The correlations between personality and environmental factors with burnout domains are presented in Table 4.

As is clear from the results presented in Table 4, all 3 burnout dimensions correlated with most of the personality factors, with some of the sociodemographic variables that were considered significant for this analysis and also both with the alexithymia dimensions and with the alexithymia total score.

Emotional exhaustion, which measures feelings of being emotionally overextended and exhausted by one's work, correlates positively with the personality factor neuroticism, with alexithymia factors (difficulty describing feelings and difficulty identifying feelings) with the alexithymia total scores and with the hours worked per week, meaning that the higher the level of these variables is, the more emotionally exhausted a person will feel. Also, emotional exhaustion correlated negatively with extraversion, which means that the more extraverted a person is, the less emotional exhaustion he or she will experience.

Depersonalization (emotional coldness and impersonal reactions to the beneficiaries of one's work) correlates positively with neuroticism, with two factors of alexithymia (difficulty describing feelings and difficulty identifying feelings), with the total score for the alexithymia scale, and with working hours per week, which means that the higher the level of neuroticism is in an individual's personality structure, the higher the levels will be for the 2 factors of alexithymia and the overall alexithymia level. The more work an individual does per week, the more depersonalization he or she will experience. Yet, depersonalization correlates negatively with all the other personality factors, extraversion, agreeableness, conscientiousness, and openness, and with the number of children that person has. This means that the more present those BFI factors are in the personality structure and the more children a person has, the less depersonalization a person will experience.

Personal accomplishment describes feelings of competence and successful achievement in one's work with people. This burnout factor correlates positively with the personality factors extraversion, agreeableness, conscientiousness, and openness. This means that the more extraverted, agreeable, conscientious, and open to new experiences a person is, the more accomplished that person will feel. Positive correlations were also identified between personal accomplishment and some of the sociodemographic variables like age, number of children, and years of experience, meaning that the older and more experienced a person becomes and the more children a person has, the more accomplished that person will feel on a personal level. On the other hand, statistically significant negative correlations were found between personal accomplishment and the BFI factor neuroticism, all the alexithymia factors (difficulty describing feelings, difficulty identifying feelings, and externally oriented thinking), the alexithymia total score, and the number of shifts per month. This means that the more neurotic a person feels, the higher his or her alexithymia level will be; the more shifts per month worked, the less accomplished a person will feel, despite all his or her other achievements. Both neuroticism and extraversion are thought to be genetically influenced [18]. The associations between depression and the dimensions of extraversion and neuroticism have been assessed in various studies [19, 20].

The more the person exhibits neuroticism as a pronounced characteristic, the more prone he or she is to feeling exhausted. Neuroticism shows the person's lability and reactivity to stressful situations.

People with conscientious structures respect rules, are perfectionists, and easily become dissatisfied. Even when it comes to common tasks, the conscientious are dissatisfied with results. Their burnout is also due to their high level of conscientiousness, not only to the task in itself, that is, to the way in which the subjects relate to the task to be completed. Our results confirm the fact that there is a strongly significant correlation between conscientiousness and values for depersonalization (negative) and personal achievement (positive).

In addition, study research proves that age tends to be positively associated with the levels of personal accomplishment.

This study shows that women are more affected by burnout than men are. Although males and females seem to be affected differently by burnout, there is no strong evidence that either sex is more at risk in studies published previously regarding burnout in physicians [21–24]. Taking into account the fact that there is very little research about burnout in obstetrics and gynecology physicians, we consider the results of this research important for this medical specialty.

### 3.4. Regression Analysis

The analysis of correlations emphasized the influence of certain personality factors and of alexithymia factors on burnout dimensions. To identify the most effective model for estimating the burnout criterion, we used multiple linear regression with the hierarchic method. Starting from this analysis, the following prediction models were established for emotional exhaustion, depersonalization, and personal accomplishment.

The linear regression analysis results presented in Table 5 show that for the emotional exhaustion criterion all the predictive models were significant. The best predictive model is model 7. With regard to the emotional exhaustion criterion, this model explains 27.5% of its variance. Among the 7 predictors of the model, only the predictors neuroticism (*p* = .000, *b* = 6.547, and beta = 0.377) and difficulty identifying feelings (*p* = .003, *b* = 0.645, and beta = 0.323) have a significant influence on emotional exhaustion, their effect being a positive one. The result proves that the more neuroticism a person shows and the more problems he or she has in identifying feelings, the higher the emotional exhaustion scores will be.

Regarding depersonalization, all the predictive models are statistically significant, but the best predictive model is also model 7. With regard to the depersonalization criterion, model 7 explains 10.2% of its variance. Among the 7 predictors of this model, only difficulty identifying feelings (*p* = .038, *b* = 0.210, and beta = 0.245) had a significant positive influence on emotional exhaustion. This result proves that the harder it is for a person to identify feelings, the higher the level of depersonalization will be.

For the personal accomplishment criterion, all the predictive models were statistically significant, but the best predictive model is model 7. For the personal accomplishment criterion, this model explains 29.4% of its variance. Among the 7 predictors of the model, none has a significant influence on personal accomplishment.

## 4. Discussions

The study explores the relationship between environmental (related to family characteristics and work context) and individual (personality factors and self-reported data) and burnout syndrome in obstetrics and gynecology physicians.

The analysis identified a high number of physicians suffering from chronic diseases related to sleep deprivation (e.g., migraines) and as a consequence of the posture adopted during medical interventions. The high number of work hours and returning to the hospital to assist patients have important negative effects on physicians' wellbeing.

The higher scores for personal achievement and depersonalization in primary physicians than in residents prove that the years of work experience and age are favorable factors for a high level in these 2 variables. The more experienced the physicians are, the more satisfaction they find in their profession. Similar results were obtained in a few studies that emphasized the differences in the level of professional satisfaction between young and experienced physicians. Although the large number of working hours, the difficulty in programming work schedule, and the high-stress work environment leave their mark on physicians' quality of life regardless of their specialty, our study shows that more experienced physicians have lower recorded levels of burnout and depersonalization and higher levels of personal achievement [25].

Though many studies prove that a large number of working hours per week are an important factor in burnout, low levels of satisfaction, and depersonalization, this study proves that these variables (and the higher number of simultaneous jobs) do not negatively influence these values. An explanation may be that many of the surveyed subjects are academics, juggling practical and theoretical activity with research. These 3 tasks become, in their turn, reasons for professional achievement, meaning that publishing articles or teaching activity, though requiring a larger number of work hours than that of physicians without teaching activity, represents a growth factor for professional satisfaction.

Unlike other medical specialties, obstetrics and gynecology requires a prompt answer to patients' demands, in that surgical interventions are more difficult to arrange, compared with other surgical specialties. An obstetrics and gynecology physician can be called to duty at any hour day or night when a patient is going into labor. Patients' lengthy labor, long shifts, or long surgical interventions may increase lack of sleep among doctors. The data obtained in our study prove that an important factor associated with burnout is insomnia, which also influences scores obtained in depersonalization and satisfaction. Lack of sleep and its consequences on the physician's physical and mental health are related to burnout.

The large number of work hours leads to an increased error rate. However, some studies have emphasized that this increased error rate caused by lack of sleep is most common in young physicians. For example, a study conducted by Grantcharov et al. [26] found that surgical residents were more prone to have double the rate of technical errors after overnight work than after a night of sleep. Our findings prove that insomnia is strongly related to burnout among obstetrics and gynecology physicians. Nonetheless, the number of hours of work is not correlated with burnout, depersonalization, or personal achievement. However, these results open the way for new research to identify whether sleep deprivation and the large number of work hours per week are variables that influence burnout only in the case of less experienced physicians.

The findings of this study are important, because no study about burnout among obstetrics and gynecology physicians has been conducted before. Our study fills an information gap about burnout related to environmental (familial and contextual characteristics) and individual (like personality traits) factors among physicians.

### 4.1. Limitations

Despite the results, which are useful for physicians, this research has some limitations. First, the physicians included in this study work in urban areas, in university hospitals, or in maternity hospitals in large cities. The access to medical facilities and the relatively small distance from patients in need could diminish professional exhaustion. Second, due to the small number of physicians who participated in the research, the results cannot be generalized. Even though the hospitals taken into account cover one-fourth of the country, the results may not be accurate for the general population of obstetrics and gynecology physicians. Third, this is a preliminary research; it does not cover all factors influencing burnout among physicians. Fourth, no investigation was performed on certain factors (such as the doctor-patient relationship and communication with superiors and colleagues) related to burnout among physicians from all specialties. The final limitation is due to the nature of this study. One of the major disadvantages of such studies is that they are limited by the fact that they are carried out at one point in time, and they do not indicate anything regarding the sequence of events, whether that factor is a cause or an outcome. This being so, it is impossible to infer causality. Even though a lot of information can be collected about potential risk factors in a cross-sectional study, these studies have a few disadvantages. With a cross-sectional study, it is difficult to make causal inferences and the research is only a snapshot; the situation may provide differing results if another time frame had been chosen.

## 5. Conclusions

Female physicians are more prone to developing emotional exhaustion than male physicians are. Insomnia and depression have a major influence on all the burnout dimensions, especially on emotional exhaustion and depersonalization, on all the alexithymia factors, and on the alexithymia total score.

Even though many things can cause burnout among obstetrics and gynecology physicians, our study shows that these physicians can experience many personal accomplishments as they grow older, as they become more experienced, as they have children, and as they work fewer shifts per month; so over time they can enjoy all the other wonderful things in their life.

Personality factors and burnout dimensions are strongly related to alexithymia factors and the alexithymia total score. The more the persons exhibit neuroticism and show reactivity to stressful situations, the more problems they will have in identifying feelings and the more emotional exhaustion they will experience. Also, the harder it is for a person to identify feelings, the higher the level of their depersonalization will be.

These results are important for obstetrics and gynecology physicians in coping with burnout. They have to take into consideration their personality traits, alexithymia domains, and environmental factors when finding strategies to adjust to their professional stress.

## Figures and Tables

**Table 1:** Sociodemographic characteristics.

Variables	*N* (%)
*Sex*	
Male	35 (30.17)
Female	81 (69.83)
*Age*	
≤30	40 (34.5)
31–40	21 (18.1)
41–50	31 (26.7)
≥51	24 (20.7)
*Level of specialization*	
Resident	42 (36.21)
Specialist	11 (9.48)
Consultant	63 (54.31)
*Work environment*	
Urban	112 (96.55)
Rural	4 (3.45)
*Marital status*	
In partnership	22 (18.97)
Married	63 (54.31)
Divorced	8 (6.90)
Single	23 (19.83)
*The partner is a doctor*	
Yes	45 (39.47)
No	59 (51.75)
*The partner had/has the same medical specialization*	
Yes	15 (13.64)
No	83 (75.45)
*The family of origin has children*	
1	38 (32.76)
2	61 (53.59)
3	9 (7.76)
>3	8 (5.89)
*Have children *	
Yes	76 (65.22)
No	40 (34.78)
*Experience as OG doctor* (Myears)	12.94 ± 10.94
*Weekly work hours*	56 ± 21
*Monthly number of shifts*	4.5 ± 2.2
*Have academic activity *	
Yes	24 (20.69)
No	92 (73.91)
*Number of simultaneous jobs*	
1	83 (71.6)
2	25 (21.6)
3	8 (6.8)
*Have chronic diseases*	
Yes	46 (39.7)
No	70 (60.3)

**Table 2:** Results for MBI, BFI, and TAS-20.

	Domains	Total	Male	Female
MBI	Emotional exhaustion	22.50 ± 12.40	18.73 ± 13.48	24.14 ± 11.71
	Depersonalization	7.18 ± 5.35	5.97 ± 5.45	7.70 ± 5.29
	Personal achievement	36.73 ± 7.82	37.76 ± 8.37	36.33 ±7.63

BFI	Extraversion	3.71 ± 0.71	3.73 ± 0.72	3.69 ± 0.71
	Neuroticism	2.79 ± 0.71	2.67 ± 0.69	2.84 ± 0.73
	Openness	3.60 ± 0.57	3.60 ± 0.61	3.61 ± 0.56
	Conscientiousness	3.99 ± 0.59	4.05 ± 0.50	3.97 ± 0.63
	Agreeableness	3.85 ± 0.83	3.80 ± 0.55	3.87 ± 0.93

TAS-20	*Difficulty identifying feelings*	15.84 ± 6.23	15.11 ± 6.44	16.17 ± 6.19
	*Difficulty describing feelings*	12.11 ± 4.05	12.32 ± 3.42	12.06 ± 4.32
	*Externally oriented thinking*	17.84 ± 4.64	18.35 ± 5.40	17.62 ± 4.33
	Total score for alexithymia	45.80 ± 11.91	45.79 ± 11.26	45.86 ± 12.31

**Table 3:** Results for MBI considering the level of specialization.

	Emotional exhaustion	Depersonalization	Personal achievement
Residents	23.41 ± 10.80	8.76 ± 5.45	33.11 ± 7.32
Specialists	19.09 ± 12.19	7.81 ± 4.72	38.45 ± 8.95
Consultants	22.49 ± 13.48	6.03 ± 5.16	38.84 ± 7.15

**Table 4:** Correlations between environmental and personality factors and alexithymia with burnout.

Variables	Emotional exhaustion	Depersonalization	Personal accomplishment
Extraversion	**R** = −****.330^**∗∗**^, **p** = .000	**R** = −****.221^**∗**^, **** **p** = .017	**R** = ****.388^**∗∗**^, **p** = .000
Agreeableness	*R* = −.158, *p* = .091	**R** = −****.313^**∗∗**^, **p** = .001	**R** = ****.368^**∗∗**^, **p** = .000
Conscientiousness	*R* = −.162, *p* = .082	**R** = −****.239^**∗∗**^, **p** = .010	**R** = .315^**∗∗**^, **p** = .001
Neuroticism	**R** = .475^**∗∗**^, **p** = .000	**R** = .231^**∗**^, **p** = .012	**R** = −.456^**∗∗**^, **p** = .000
Openness	*R* = −.148, *p* = .112	**R** = −.218^**∗**^, **p** = .019	**R** = .363^**∗∗**^, **p** = .000
Difficulty describing feelings	**R** = .205^**∗**^, **p** = .028	**R** = ****.238^**∗**^, **p** = .010	**R** = −.370^**∗∗**^, **p** = .000
Difficulty identifying feelings	**R** = ****.391^**∗∗**^, **p** = .000	**R** = .321^**∗∗**^, **p** = .000	**R** = −.440^**∗∗**^, **p** = .000
Externally oriented thinking	*R* = .081, *p* = .391	*R* = .165, *p* = .078	**R** = −.206^**∗**^, **p** = .027
Total score for alexithymia	**R** = ****.314^**∗∗**^, **p** = .001	**R** = ****.278^**∗∗**^, **p** = .003	**R** **** = −****.408^**∗∗**^, **p** = .000
Age	*R* = .073, *p* = .438	*R* = −.155, *p* = .096	**R** = ****.358^**∗∗**^, **p** = .000
Number of children	*R* = −.103, *p* = .274	**R** = −.274^**∗∗**^, **p** = .003	**R** = .216^**∗**^, **p** = .020
Experience in years	*R* = −.043, *p* = .645	*R* = −.124, *p* = .187	**R** = .352^**∗∗**^, **p** = .000
Working hours/week	**R** = .305^**∗∗**^, **p** = .001	**R** = .262^**∗∗**^, **p** = .006	*R* = −.060, *p* = .536
Shifts/month	*R* = −.012, *p* = .897	*R* = .156, *p* = .095	**R** = −.186^**∗**^, **p** = .045

^*∗∗*^ *p* < .01 and ^*∗*^*p* < .05.

**Table 5:** Regression analysis.

	Models		Emotional exhaustion	Depersonalization	Personal accomplishment
Step 1	Extraversion	*R* ^2^ adjusted	0.100	0.029	0.152
		Δ*R*^2^	0.108	0.037	0.160
		*F*	13.621^**∗∗**^	4.371^**∗**^	21.450^**∗∗**^

Step 2	Extraversion, agreeableness	*R* ^2^ adjusted	0.099	0.047	0.183
		Δ*R*^2^	0.007	0.027	0.038
		*F*	7.271^**∗∗**^	3.825^**∗**^	13.748^**∗∗**^

Step 3	Extraversion, agreeableness, conscientiousness	*R* ^2^ adjusted	0.094	0.066	0.204
		Δ*R*^2^	0.003	0.027	0.028
		*F*	4.958^**∗∗**^	3.690^**∗**^	10.761^**∗∗**^

Step 4	Extraversion, agreeableness, conscientiousness, neuroticism	*R* ^2^ adjusted	0.226	0.064	0.236
		Δ*R*^2^	0.135	0.006	0.037
		*F*	9.309^**∗∗**^	2.933^**∗**^	9.793^**∗∗**^

Step 5	Extraversion, agreeableness, conscientiousness, neuroticism, openness	*R* ^2^ adjusted	0.221	0.060	0.261
		Δ*R*^2^	0.002	0.005	0.031
		*F*	7.642^**∗∗**^	2.451^**∗**^	9.073^**∗∗**^

Step 6	Extraversion, agreeableness, conscientiousness, neuroticism, openness, difficulty describing feelings	*R* ^2^ adjusted	0.218	0.073	0.281
		Δ*R*^2^	0.004	0.021	0.025
		*F*	6.305^**∗∗**^	2.506^**∗**^	8.436^**∗∗**^

Step 7	Extraversion, agreeableness, conscientiousness, neuroticism, openness, difficulty describing feelings, difficulty identifying feelings	*R* ^2^ adjusted	0.275	0.102	0.294
		Δ*R*^2^	0.060	0.035	0.018
		*F*	7.183^**∗∗**^	2.847^**∗∗**^	7.785^**∗∗**^

Step 8	Extraversion, agreeableness, conscientiousness, neuroticism, openness, difficulty describing feelings, difficulty identifying feelings, externally oriented thinking	*R* ^2^ adjusted	0.268	0.096	0.288
		Δ*R*^2^	0.000	0.003	0.001
		*F*	6.229^**∗∗**^	2.519^**∗**^	6.764^**∗∗**^

^*∗∗*^ *p* < .01 and ^*∗*^*p* < .05.
